# Patient-derived xenograft models—the future of personalised cancer treatment

**DOI:** 10.1038/s41416-019-0678-0

**Published:** 2020-01-10

**Authors:** Jenna Bhimani, Katie Ball, Justin Stebbing

**Affiliations:** 1 Department of Oncology, Charing Cross Hospital, Imperial College and Imperial College Healthcare NHS Trust, Fulham Palace Road, London, W6 8RF UK; 2 Imperial College Centre for Translational and Experimental Medicine, Hammersmith Hospital, Du Cane Road, London, W12 0NN UK

## Abstract

For many tumours there is a lack of randomised data from which we can guide systemic treatments. Although gene expression profiling along with proteomics has led to advances in diagnosis, classification and prognosis, our ability to target many cancers has been further limited due to a lack of therapeutic options. The use of patient-derived xenograft (PDX) models in the setting of a rare malignancy is discussed here by Kamili et al, with the successful establishment of new model systems.

## Main

There is almost always a discrepancy between preclinical efficacy in trials and actual clinical outcomes. This generates a demand for improving preclinical modelling. The rapidly evolving field of targeted personalised therapy is the future of oncological practice and sometimes this cannot be evaluated through traditional research methodology, such as randomised control trials (RCTs).^1^ Biomarker-driven therapy has become integral to treatment of cancer patients, leading to the introduction of novel trial designs with populations of biomarker-identified patient groups.^2^ A review of predictive and prognostic tumour biomarkers advocated that reliance on clinical judgement and expertise is vital in developing personalised cancer medicine, rather than utilisation of published clinical data.^3^

Despite the variability and heterogeneity of cancer types, most treatments remain ‘generic’ and usually involve chemotherapy as the mainstay.^4^ Chemotherapy has often been shown to be only minimally beneficial to overall survival, and is often ineffective with intolerable side effects—though one could argue that this is the case with all treatments. The hidden costs of managing chemotherapy toxicities, with repeated admissions and discussions about side effects, are likely to be substantial. Targeted monoclonal antibodies, immune checkpoint inhibitors and CAR T-cell therapies have shown promising advances in individualised cancer treatment, however, only a few are available for standard clinical practice.^4^ In Phase 1 clinical trials, both response rate and progression-free survival were greatly improved with personalised oncology therapy by using biomarker selection strategies compared with those undergoing generic treatment.^5^ Currently, developments in the detection of cancer drug targets, comprehensive molecular profiling and personalised combined treatment regimens are all contributing to increasing availability of personalised oncology to a wider range of patients.^6^

Patient-derived xenograft (PDX) models have been increasingly used in translational research since their development.^7^ Currently, cell-line xenografts are the standard for preclinical research, able to create a tumour microenvironment.^8^ More often than not however, they do not accurately reflect the true behaviour of the host tumour and are able to adapt to in vitro growth, losing the original properties of the host tumour.^7^ Other models for tumour graft strategies have previously had limited success.^8^

Trials examining PDX models have shown that they can produce samples that are authentic to the host tumour.^9^ They are able to accurately replicate tumour growth, diversity of tumour cells and tumour progression, including metastatic potential.^7,9,10^ PDX models have been shown to be used for prognostication: studies have shown that successful engraftment is associated with a poorer prognosis that can be correlated clinically.^10^ Another study, by exploring heterogeneous sarcoma patients with a wide range of prognoses and tumour subtypes, demonstrated that PDX models aided therapeutic decision-making in the case of a collection of disparate tumours where each one is a rare subtype.^11^

While neuroblastomas are the most common extracranial solid tumour in children, they are generally rare, and have a wide variety of outcomes depending on the specific, albeit variable, biology of the tumour.^12–14^ Children with high-risk neuroblastoma have a less than 50% chance of cure.^10^ This has led to recognition of an increasing need for personalised treatment for patients with high-risk neuroblastomas.^15^ This is especially relevant as current treatment regimens have a range of acute toxicities and long-term side effects.^15,16,17^ RCTs effectively test new interventions, remove allocation bias, are ethically conducted and ensure that no subject receives less-than-baseline care; however, they are expensive and can take many years to complete. RCTs are also ultimately not appropriate for the requirement for rapid developments in any field, let alone a rare cancer^18^—where they are less valid, almost impossible to recruit adequate numbers for and are thus performed less frequently, leading to a dearth of evidence.^19^ In children there is a scarcity of trials conducted, with issues relating to feasibility as well as ethics. This leads to an absence of evidence, and alternatives need to be sought.^20^ Existing in vivo and in vitro data will always remain the preclinical vanguards of drug development, but the clinical use of patient-derived xenografts can enhance the robustness of preclinical studies.^14^

In this edition of the BJC, Kamili et al. investigated the reliability of establishing PDX models for high-risk neuroblastomas.^16^ They examine different techniques including different engraftment sites and different biological sample types, such as metastatic and primary tumour samples. Previous papers have reported that PDX models are more informative than cell-line xenografts; however, they have demonstrated limited engraftment success rates, prolonged establishment of grafts and high costs.^12,13,21^ This paper’s key finding is that of successful engraftment via orthotopic implantation, a method leading to more rapid model development. This is in keeping with previous research in advanced sarcoma patients, which highlighted the need for time-efficient engraftment in order to see a benefit within a clinically appropriate timescale.^16^

Kamili et al. are able to address the lengthy establishment time that has been a limiting factor in previous studies.^16^ All of the orthotopically inoculated tumours were successfully engrafted and resulted in the quickest time to engraftment compared with subcutaneously and intramuscularly inoculated tumours. Indeed, PDX models were established for 4 of 9 of patients at diagnosis and all patients tested (5/5) at relapse.^16^ These findings show that PDX can be established quickly, which is key in high-risk and rapidly developing tumours. Although the orthotopic model was found to be the most rapid engraftment approach, there were also subcutaneous and intramuscular xenografts that were found to be equally as representative of the donor tumour. This suggests that in future, the xenograft type can be selected depending on its clinical utility. It has also shown that PDX models can reliably be established from a diverse range of samples, depending on which is most accessible clinically.^16^ This is going to be especially central to rare cancers, paediatric tumours or those with a range of subtypes, though one can argue that oncology is heading in that direction already. It is also recognised that tissue samples from tumours have often been inaccessible, and this has been a limiting factor.^21^ This is significant for development of personalised models that can be used in future clinical trials and clinical practice. Another important aspect of this paper is the expansion of patient material for ex vivo and in vivo drug testing. Personalised PDX models would allow for prioritisation of therapeutic options and provide an evidence-based platform for decisions regarding personalised therapy.^16^

This research explores the challenges of developing PDX models, including xenogeneic graft versus host disease and proliferation of EBV-infected cells. It does, however, propose a strategy to overcome and limit using this T-lymphocyte depletion.^16^ It emphasises the possibility that a personalised approach to cancer treatment and research can be developed with these models. With the development of reliable engraftment, this could lead to informative preclinical models for individual patients.^16,22^ It also proposes the use of xenografts to expand the current limited basis for drug testing in cancer patients, minimising the need for expensive and prolonged randomised controlled trials.^7,9^ Changes in research practice are needed to adapt to the current medical climate. Innovations, such as PDX models, have the ability to change what is considered standard practice and improve access to personalised treatment.

## Data Availability

Not applicable.
